# Determination of Mineral Constituents, Phytochemicals and Antioxidant Qualities of *Cleome gynandra*, Compared to *Brassica oleracea* and *Beta vulgaris*

**DOI:** 10.3389/fchem.2017.00128

**Published:** 2018-01-04

**Authors:** Mack Moyo, Stephen O. Amoo, Adeyemi O. Aremu, Jiri Gruz, Michaela Šubrtová, Monika Jarošová, Petr Tarkowski, Karel Doležal

**Affiliations:** ^1^Department of Horticultural Sciences, Faculty of Applied Sciences, Cape Peninsula University of Technology, Bellville, South Africa; ^2^Agricultural Research Council, Roodeplaat Vegetable and Ornamental Plants, Pretoria, South Africa; ^3^Indigenous Knowledge Systems Centre, Faculty of Natural and Agricultural Sciences, North-West University, Mmabatho, South Africa; ^4^Laboratory of Growth Regulators & Department of Chemical Biology and Genetics, Centre of the Region Haná for Biotechnological and Agricultural Research, Faculty of Science, Palacký University & Institute of Experimental Botany AS CR, Olomouc, Czechia; ^5^Centre of the Region Haná for Biotechnological and Agricultural Research, Central Laboratories and Research Support, Faculty of Science, Palacký University, Olomouc, Czechia; ^6^Centre of the Region Haná for Biotechnological and Agricultural Research, Department of Genetic Resources for Vegetables, Medicinal and Special Plants, Crop Research Institute, Olomouc, Czechia

**Keywords:** free radicals, indigenous leafy vegetables, minerals, phenolic acids, vitamins

## Abstract

The study compared mineral, chemical and antioxidant qualities of *Cleome gynandra*, a wild leafy vegetable, with two widely consumed commercial vegetables, *Brassica oleracea* and *Beta vulgaris*. Mineral nutrients were quantified with inductively coupled plasma mass spectrometry (ICP-MS), phenolic compounds using ultra-high performance liquid chromatography coupled to a mass spectrometer (UHPLC-MS) and β-carotene and vitamin C using high performance liquid chromatography with a photodiode array detector (HPLC-PDA). The antioxidant potential was evaluated using 2,2–diphenyl−1–picryl hydrazyl (DPPH) and oxygen radical absorbance capacity (ORAC) assays. *Cleome gynandra* had highest concentrations of phosphorus, potassium, calcium, iron, zinc, ascorbic acid, total phenolics, and flavonoids; whereas sodium, magnesium, manganese, copper and β-carotene were higher for *B. vulgaris*. The significantly higher antioxidant activity (*P* ≤ 0.05) exhibited by *C. gynandra* in comparison to the two commercial vegetables may be due to its significantly high levels of vitamin C and phenolic acids. These findings on the mineral, chemical and antioxidant properties of *C. gynandra* provide compelling scientific evidence of its potential in adding diversity to the diet and contributing toward the daily nutritional requirements of millions of people for food and nutritional security.

## Introduction

*Cleome gynandra* L. (common names: Shona cabbage, spider plant, African cabbage or cat's whiskers) is a widely consumed wild leafy vegetable, which belongs to the Cleomaceae family. It is an erect and branched herbaceous plant, which grows wild in southern Africa (van Rensburg et al., 2004), East Africa as well as South Asia (Bala et al., 2010; Cernansky, 2015). *C. gynandra* is traditionally harvested from the veld or semi-cultivated in sub-Saharan Africa (van Rensburg et al., 2004; Habwe et al., 2009; Gao et al., 2011). Besides its nutritional benefits, *C. gynandra* has anti-bacterial (Hamill et al., 2003), anti-inflammatory (Narendhirakannan et al., 2007), antioxidant (Muchuweti et al., 2007), and anticancer (Bala et al., 2010) properties. The nutritional qualities and biological activities of *C. gynandra* are largely attributed to its “large amounts” of vitamins and minerals (Gao et al., 2011), and its diversity of chemical constituents, which include tannins, saponins, alkaloids, steroids, glycosides, flavonoids, and phenolic compounds (Narendhirakannan et al., 2007). Based on its high visibility in scientific literature, *C. gynandra* is fast emerging as one of the most widely consumed “super vegetables” (Cernansky, 2015), especially in sub-Saharan Africa and Asia.

For millennia, communities throughout the African continent have relied on natural plant resources for life's necessities, most notably food and medicine. In particular, they have used indigenous leafy vegetables as a source of vitamins, dietary mineral nutrients and for general well-being maintenance (Uusiku et al., 2010). The consumption of traditional dishes is a global phenomenon: for example, wild food plants were recently reported to constitute a significant part of diets across Europe (Guarrera and Savo, 2016). Worldwide, it is estimated that 7,000 plant species are semi-cultivated or harvested from the wild for food (Schönfeldt and Pretorius, 2011). The characteristics of indigenous leafy vegetables, which make them attractive, include their ability to grow on low fertility soils, high relative drought tolerance and faster growth rates; hence, they can be harvested within a short period of time (van Jaarsveld et al., 2014). Despite being in the scientific doldrums, this important food source has gradually evolved over many generations of continuous local scale innovation. It is only recently that scientists have awakened to the promising potential of indigenous leafy vegetables as sources of nutrition in enhancing food security and maintaining healthy lifestyles. Although there are documented studies conducted on some individual vegetables, a comparison of nutrient content results from different data sources could be problematic due to variations in sample preparation and analytical conditions used in different studies (van Jaarsveld et al., 2014).

The objective of the current study was to compare the mineral composition, chemical constituents and antioxidant activity of *C. gynandra* against two of the most widely-consumed commercial vegetables in the world; *viz Brassica oleracea* var. *capitata* (cabbage, Brassicaceae) and *Beta vulgaris* L. (Swiss chard, Amaranthaceae). Cabbage (*Brassica oleracea* var. *capitata*) is regarded as one the world's leading vegetable crops that is rich in vitamins, anticarcinogenic glucosinolates, and antioxidant metabolites as well as amino acids (Rengasamy et al., 2016). On the other hand, Swiss chard (*B. vulgaris* L.) spread from Europe, where it has been cultivated since classical antiquity, to the rest of the world (Pyo et al., 2004). Coupled to its nutritional characteristics, *B*. *vulgaris* has been demonstrated to possess anti-acetylcholinesterase, anti-inflammatory and antioxidant properties (Ninfali et al., 2007).

## Materials and methods

### Plant materials

Fresh *Brassica oleracea* var. *capitata* cv. Drumhead and *Beta vulgaris* L. cv. Fordhook Giant were purchased from local supermarkets (Pick n Pay and Checkers), Pietermaritzburg, South Africa. Fresh *C. gynandra* was purchased from Mbare market, Harare, Zimbabwe. Plant material was lyophilized using a freeze-dryer (VirTis BenchTop Pro with Omnitronics™, SP Scientific) and ground to fine powder.

### Mineral analysis using ICP-MS

The freeze-dried vegetable samples were digested in diffused microwave system (MLS 1200 Mega; Milestone S.r.L., Sorisole, Italy) following the description by Jarošová et al. (2014) with slight modifications. In triplicates, the samples (about 15–25 mg) were weighed into polytetrafluoroethylene vessels and 2 ml of HNO_3_ (67%, analpure) and 1 ml of H_2_O_2_ (30%, analytical grade) (both Analytika Ltd., Prague, Czech Republic) were added. After the digestion, each solution was diluted to 15 ml in a test tube with deionised water and analyzed by ICP-MS.

The analyses were carried out using an ICP-MS (Agilent 7,700x; Agilent Technologies, Tokyo, Japan) based on quadrupole mass analyser and octapole reaction system (ORS 3). Collision cell in He-mode was used for elimination of possible polyatomic interferences and instrument was set up by using Tuning solution (Agilent Technologies, Santa Clara, USA). Isotopes ^23^Na and ^24^Mg were measured in a gas mode whereas isotopes ^31^P, ^39^K, ^44^Ca, ^55^Mn, ^56^Fe, ^63^Cu, and ^66^Zn were measured in He-mode. ^6^Li, ^45^Sc, and ^74^Ge were used as internal standards. The calibration solutions were prepared by the appropriate dilution of the single element certified reference materials with 1.000 ± 0.002 g/l for each element (Analytika Ltd., Prague, Czech Republic) with deionised water (18.2 MΩ·cm, Direct-Q; Millipore, Molsheim, France). The certified reference materials of strawberry leaves and green algae (METRANAL® 3 and METRANAL® 8, Analytika Ltd., Prague, Czech Republic) were used for controlling decomposition process and for method validation. Measurement accuracy was verified by using certified reference material of water TM-15.2 (National Water Research Institute, Ontario, Canada).

### Quantification of total phenolics and flavonoids

Following the extraction method described by Amoo et al. (2012), the determination of total phenolic content for the three vegetable samples was performed using the Folin and Ciocalteu method (Singleton and Rossi, 1965) with slight modifications as outlined by Fawole et al. (2009). Gallic acid was used as the standard for plotting the calibration curve. Total phenolic content was expressed in mg gallic acid equivalents (GAE) per g dry weight (DW).

The flavonoid content of the three vegetable samples was quantified using the aluminum chloride colorimetric method as described by Zhishen et al. (1999). Catechin was used as a standard for the calibration curve and total flavonoid content was expressed in mg catechin equivalents (CE) per g DW.

### Determination of β-carotene

β-Carotene extraction and quantification using HPLC-PDA were done as described by Biehler et al. (2010) with modifications. In brief, samples were extracted (0.1 g/ml) with ice-cold hexane: acetone (1:1, v/v). The mixture was vortexed for 2 min before centrifuging at 2,000 rpm for 2 min. The organic phase was decanted into a tube containing saturated sodium chloride solution and placed on ice. The remaining residue was similarly re-extracted until the extract is colorless. Each time, the extracts were combined in saturated sodium chloride solution tube. The separated organic phase was filtered through a 0.45 μm syringe filter before injection into HPLC. The analysis was carried out on Prominence-*i* HPLC-PDA model system equipped with sample cooler LC-2030C (Shimadzu, Kyoto, Japan). Chromatographic separation was achieved using a C_18_ Luna® column (150 × 4.6 mm, 5 μ) maintained at 35°C. An isocratic mobile phase which consisted of acetonitrile: dichloromethane: methanol (7:2:1) was used with a flow rate of 1 ml/min, an injection volume of 20 μl and the detection at 450 nm. Peak identification and quantification were achieved based on authentic β-carotene standard, which was used for plotting the calibration curve.

### Quantification of ascorbic acid

The method described by Odriozola-Serrano et al. (2007) and Parbhunath et al. (2014) was followed with slight modifications. Individual sample was weighed (1 g) into a tube, followed by the addition of 5% metaphosphoric acid (10 ml). It was sonicated in ice-cold water bath for 15 min before centrifuging and filtration. The analysis was carried out on Prominence-*i* HPLC-PDA model system described above. Chromatographic separation was achieved using a C_18_ Luna® column (150 × 4.6 mm, 5 μl) maintained at 25°C. An isocratic mobile phase made up of water: acetonitrile: formic acid (99:0.9:0.1) at a flow rate of 1 ml/min was used. The injection volume was 20 μl and the detection was set at 245 nm. Sample quantification was achieved based on the calibration curve plotted using L-ascorbic acid.

### Quantification of individual phenolic acids

Freeze-dried samples of the three vegetables were homogenized with 80% methanol (40 mg/ml) in a 1.5 ml Eppendorf tube, using an oscillation ball mill (MM 301, Retsch, Haan, Germany) at a frequency of 25 Hz for 3 min. Deuterium-labeled internal standards were added to the extraction solvent prior to plant material homogenization. The extracts were centrifuged for 10 min at 26,000 g and the supernatant was filtered through 0.45 μm nylon microfilters (Alltech, Breda, Netherlands). The concentration of phenolic acids in vegetable extracts was determined using UHPLC (Waters, Milford, MA, USA) linked to a Micromass Quattro micro® API benchtop triple quadrupole mass spectrometer (Waters MS Technologies, Manchester, UK) as originally described by Gruz et al. (2008). The analyses were performed using three replicates per sample.

### 2,2–Diphenyl−1–picryl hydrazyl (DPPH) free radical scavenging activity

The determination of free radical scavenging activity of the three vegetable extracts was carried out as described by Amoo et al. (2012) using freshly prepared methanolic DPPH (100 μM). Decrease in the purple colouration of the reaction mixtures was read at 517 nm using a UV/VIS Specord 210 plus (Analytik Jena, Germany) spectrophotometer. Ascorbic acid was used as a standard antioxidant. Methanol, which was used for extraction, served as the negative control. The assay was performed in triplicate. The free radical scavenging activity (RSA) of the vegetable extracts was calculated according to the formula:

$$\text{RSA}(\%)=100\times (1-A_{\text{E}}/A_{\text{D}})$$

where A_E_ is the absorbance of the reaction mixture containing the sample extract or standard antioxidant, and A_D_ is the absorbance of the negative control.

### Oxygen radical absorbance capacity (ORAC)

The oxygen radical absorbance capacity (ORAC) was measured as described by Ou et al. (2001). Fluorescein (100 μl, 500 mM) and vegetable extracts (25 μl) were added into each working well in a 96-well microplate and shaken. The reaction was initiated by the addition of AAPH (25 μl, 250 mM) pre-incubated at 37°C. The fluorescence (Ex. 485 nm, Em. 510 nm) was read every 3 min over 90 min in a microplate reader Infinite M200 Pro (Tecan, Switzerland) incubated at 40°C. The net area under the curve was used to calculate antioxidant capacity in trolox equivalents (μmol TE/g). The analysis was carried out in triplicate.

### Data analysis

Statistical significance was determined using one-way analysis of variance (ANOVA) followed by a post hoc test (Duncan's multiple range or Tukey's multiple comparison tests). Data on total phenolic content, total flavonoid content, β-carotene, ascorbic acid and DPPH free radical scavenging activity were subjected to ANOVA followed by Tukey's post hoc test using GraphPad Prism version 5.02 (GraphPad Software Inc., San Diego, USA). SPSS version 16 (SPSS Inc., Chicago, IL, USA) was used to evaluate significant differences in the concentrations of phenolic acids. Differences in phenolic acid concentrations were further separated using Duncan's multiple range test. All analyses were done at a probability of α = 0.05. Normality of residuals and equality of variance were tested using the Kolmogorov-Smirnov and Levene's tests (SPSS version 16). Percentage data were arcsin transformed prior to being subjected to ANOVA.

## Results

### Mineral element composition

**Figure 1:**
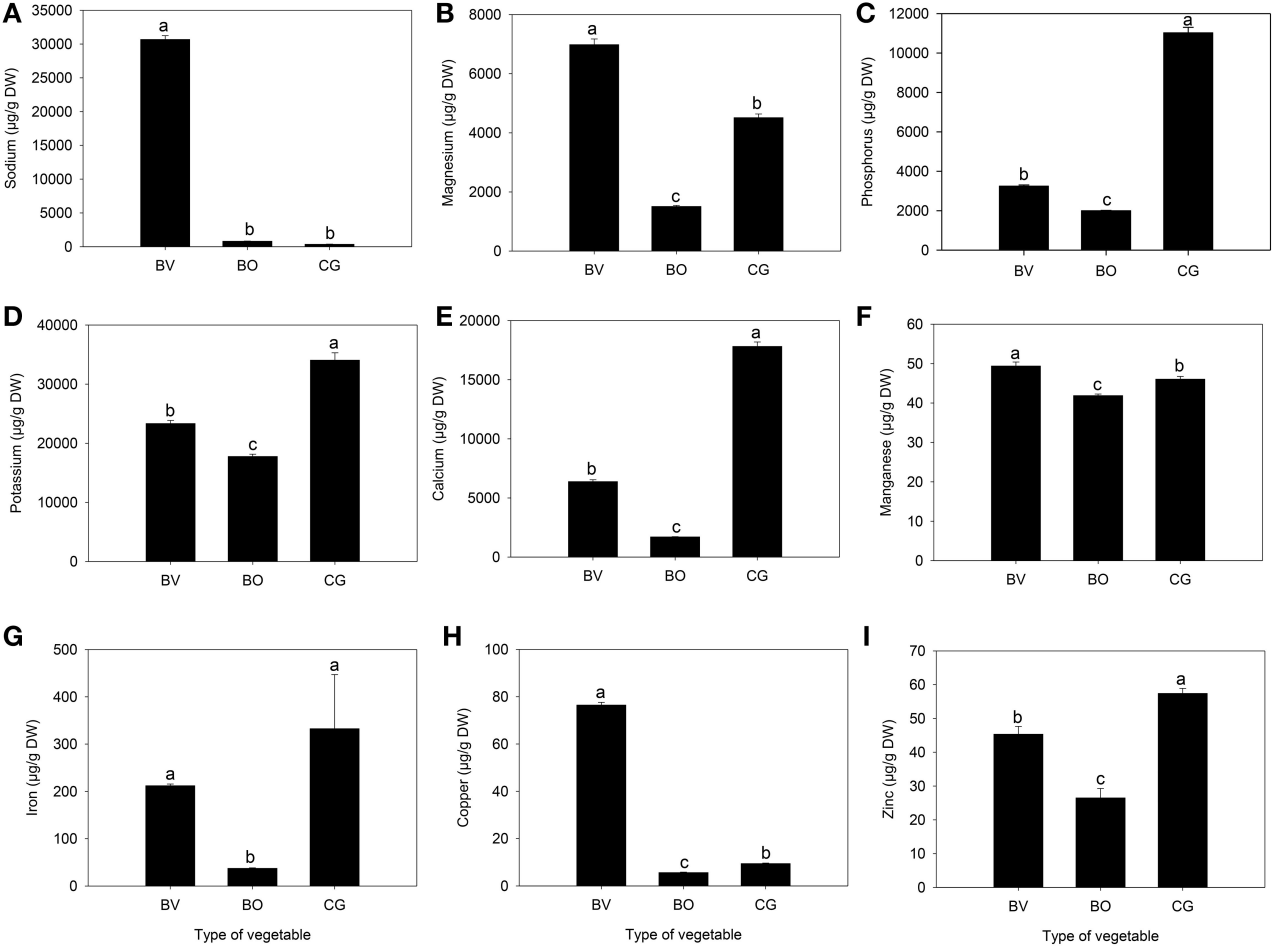
The concentration (μg/g DW) of different nutrient elements quantified in *Beta vulgaris* (BV), *Brassica oleracea* (BO) and *Cleome gynandra* (CG). **(A)** Sodium; **(B)** Magnesium; **(C)** Phosphorus; **(D)** Potassium; **(E)** Calcium; **(F)** Manganese; **(G)** Iron; **(H)** Copper; **(I)** Zinc. Data are mean ± standard error (*n* = 3). In each graph, bars with different letter are significantly different based on Tukey's test (*P* ≤ 0.05). Data analysis was performed using SPSS (version 16).

Different concentrations of macro (magnesium, phosphorus, potassium, calcium) and micro (sodium, manganese, iron, copper, and zinc) elements were identified and quantified in *C. gynandra, B. vulgaris* and *B. oleracea* vegetable extracts (Figure 1). Compared to *B. oleracea, C. gynandra* had a significantly higher content of all the quantified mineral nutrients except sodium. Overall, *B. vulgaris* exhibited significantly high concentrations of sodium, magnesium, manganese and copper. On the other hand, the content of phosphorus, potassium, calcium, iron and zinc were significantly higher in *C. gynandra* compared to both *B. vulgaris* and *B. oleracea*. It is particularly noteworthy that the concentration of phosphorus in *C. gynandra* was 3.3 and 5.5 times greater than that of *B. vulgaris* and *B. oleracea*, respectively. Similarly, the calcium content in *C. gynandra* was 2.7-fold more than in *B. vulgaris* and 10.4-fold higher than in *B. oleracea*. Notwithstanding, *B. vulgaris* had 82, 8.4, and 1.5 times greater concentration of sodium, copper and magnesium compared to *C. gynandra*. The content of zinc in *C. gynandra* was twice that of *B. oleracea*.

### Chemical constituents

**Figure 2:**
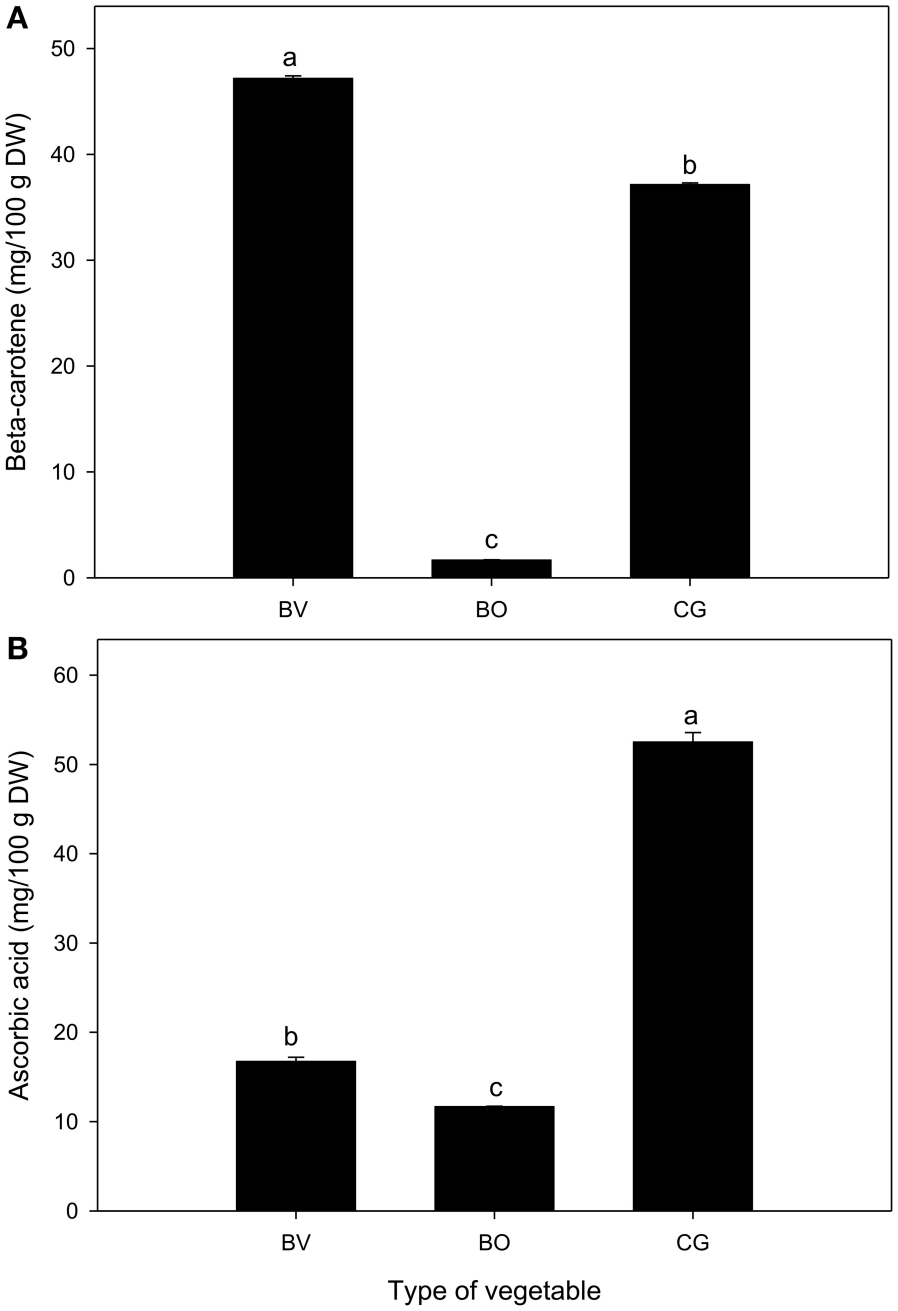
Nutritional content (mg/100 g DW) of *Beta vulgaris* (BV), *B. oleracea* (BO) and *Cleome gynandra* (CG). **(A)** β-carotene content; **(B)** Ascorbic acid content. Data are mean ± standard error (*n* = 3). In each graph, bars with different letter are significantly different based on Tukey's test (*P* ≤ 0.05). Data analysis was performed using GraphPad Prism (Version 5.02).

The total phenolic content (mg GAE/g DW) of *C. gynandra* was significantly higher than that of *B. vulgaris* and *B. oleracea* (Table 1). A similar trend was observed for total flavonoid content (CE/g DW). However, β-carotene content (mg/100 g DW) was significantly greatest in *B. vulgaris*, followed by *C. gynandra* and *B. oleracea* (Figure 2A). The concentration of β-carotene was 21.9 times higher in *C. gynandra* compared to *B. oleracea*. On the other hand, the concentration of ascorbic acid (mg/100 g DW) was observed to significantly decrease in the order: *C. gynandra* > *B. vulgaris* > *B. oleracea* (Figure 2B). In particular, the concentration of ascorbic acid in *C. gynandra* was 3.2- and 4.7-fold higher than that of *B. vulgaris* and *B. oleracea*, respectively.

**Table 1:** Total phenolics, flavonoid content and 2,2–diphenyl−1–picryl hydrazyl (DPPH) free radical scavenging activity of *Beta vulgaris, B. oleracea* and *Cleome gynandra* extracts.

**Type of vegetable**	**Total phenolics (mg GAE/g DW)**	**Flavonoids (mg CE/g DW)**	**Free radical scavenging activity (%)**		
			**200 μg/ml**	**50 μg/ml**	**3.125 μg/ml**
*Beta vulgaris*	11.04 ± 0.19*b*	2.30 ± 0.17*b*	57.54 ± 0.94*b*	18.60 ± 0.64*b*	3.07 ± 0.41*b*
*Brassica oleracea*	5.84 ± 0.65*c*	0.99 ± 0.02*c*	16.86 ± 1.38*c*	5.50 ± 0.65*c*	2.70 ± 0.35*b*
*Cleome gynandra*	15.15 ± 0.52*a*	5.65 ± 0.30*a*	80.64 ± 0.36*a*	47.69 ± 1.17*a*	8.37 ± 0.22*a*

*CE = catechin equivalents; DW = dry weight; GAE = gallic acid equivalents. Data are mean ± standard error (n = 3). Mean values within each column followed by different letter are significantly different (P ≤ 0.05) based on Tukey's Multiple Comparison Test. Data analysis was performed using GraphPad Prism Version 5.02*.

**Figure 3:**
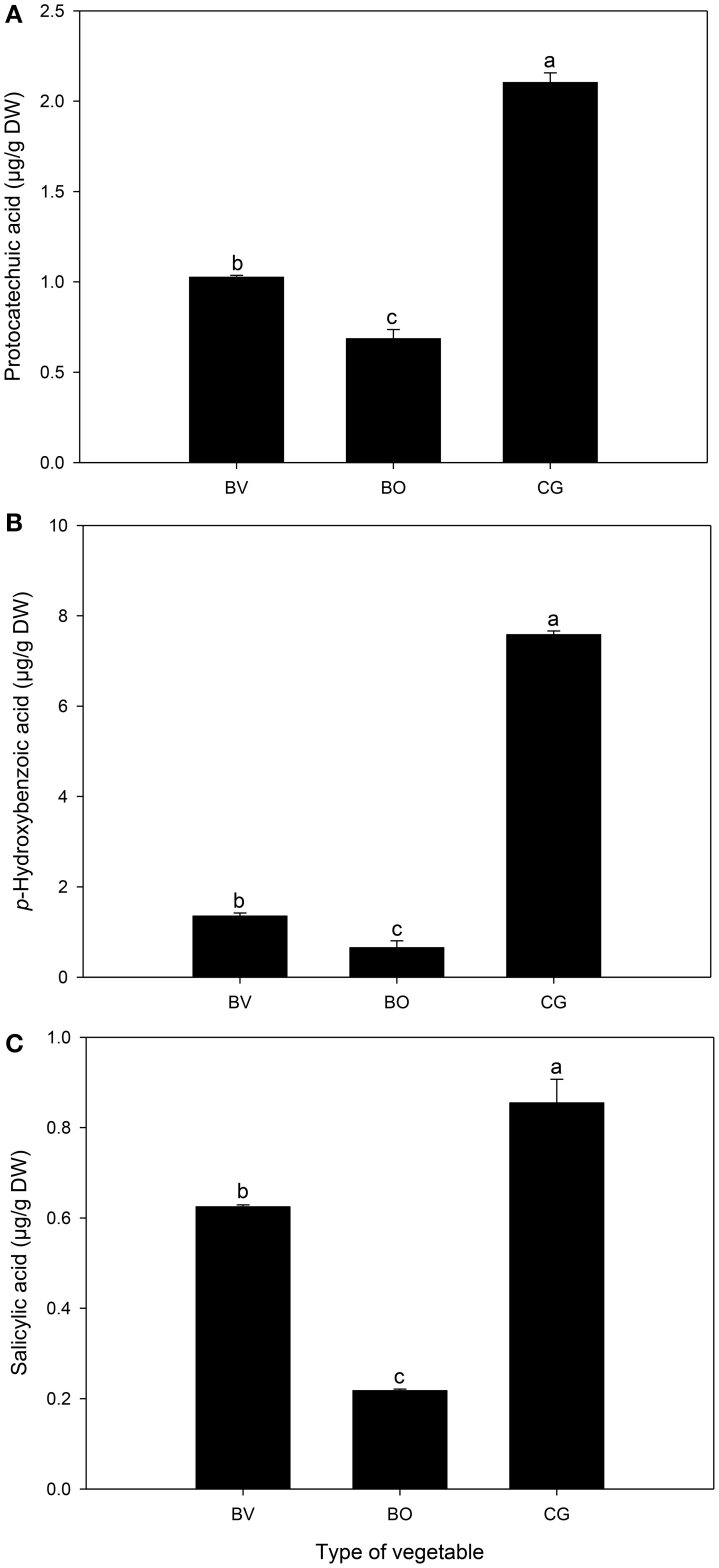
The concentration (μg/g DW) of different hydroxybenzoic acid derivatives detected and quantified in *Beta vulgaris* (BV), *B. oleracea* (BO) and *Cleome gynandra* (CG). **(A)** Protocatechuic acid; **(B)** *p*-Hydroxybenzoic acid; **(C)** Salicylic acid. Data are mean ± standard deviation (*n* = 3). In each graph, bars with different letter are significantly different based on Duncan's multiple range test (*P* ≤ 0.05). Data analysis was performed using SPSS (Version 16).

**Figure 4:**
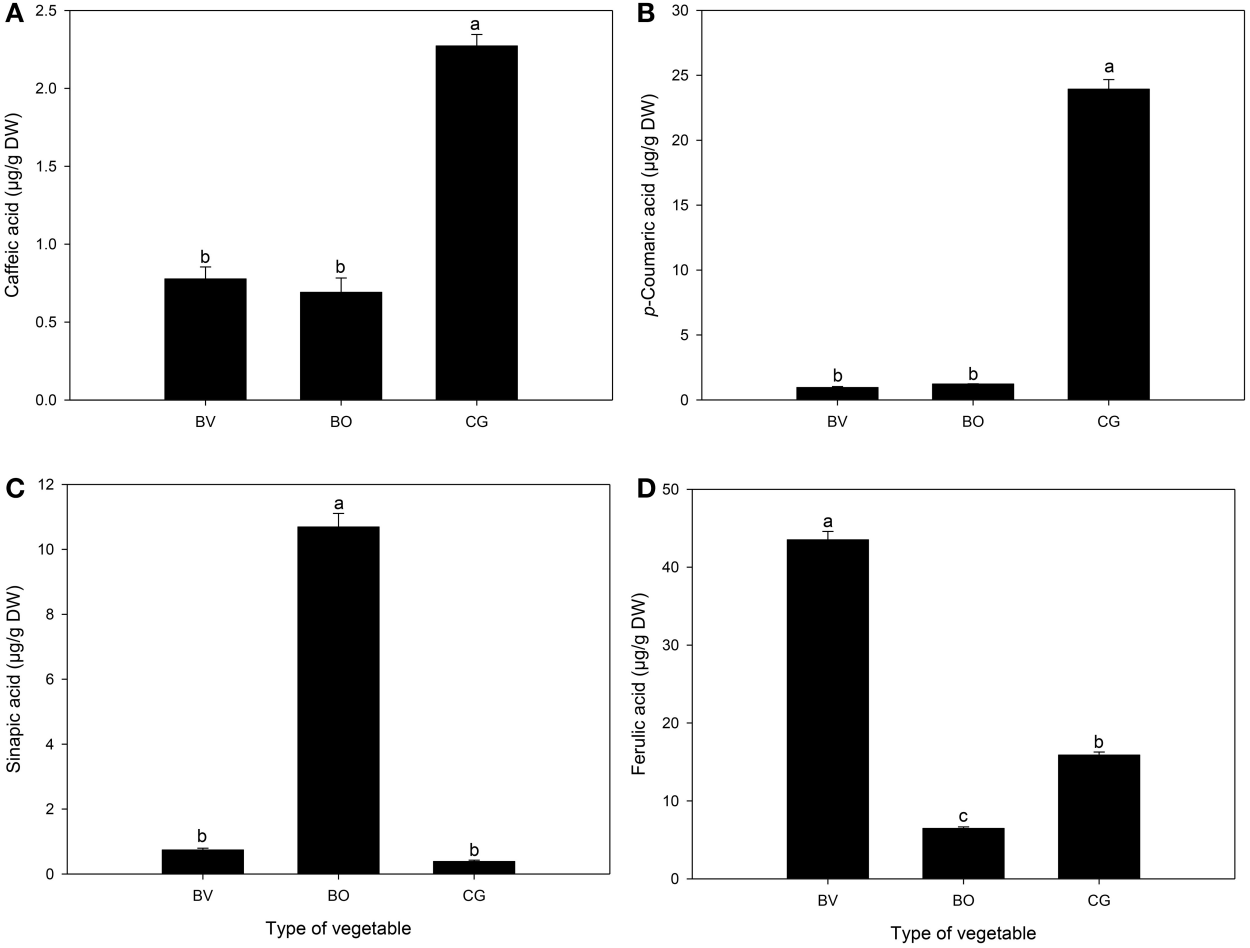
The concentration (μg/g DW) of different hydroxycinnamic acid derivatives detected and quantified in *Beta vulgaris* (BV), *B. oleracea* (BO) and *Cleome gynandra* (CG). **(A)** Caffeic acid; **(B)** *p*-Coumaric acid; **(C)** Sinapic acid; **(D)** Ferulic acid. Data are mean ± standard error (*n* = 3). In each graph, bars with different letter are significantly different based on Duncan's multiple range test (*P* ≤ 0.05). Data analysis was performed using SPSS (Version 16).

Varying concentrations of both hydroxybenzoic acids (Figure 3) and hydroxycinnamic acids (Figure 4) were identified and quantified in the three vegetables. Compared to *B. vulgaris* and *B. oleracea, C. gynandra* exhibited significantly high concentrations of protocatechuic acid, *p*-hydroxybenzoic acid and salicylic acid. The content of *p*-hydroxybenzoic acid was 11- and 6-folds higher in *C. gynandra* compared to *B. oleracea* and *B. vulgaris*, respectively. For protocatechuic acid and salicylic acid, the concentration in *C. gynandra* was at least 2 times greater than that of *B. vulgaris* and *B. oleracea*. The identified and quantified hydroxycinnamates were caffeic acid, *p*-coumaric acid, sinapic acid, and ferulic acid (Figure 4). The concentration of caffeic acid (2.27 μg/g DW) and *p*-coumaric acid (23.9 μg/g DW) was significantly high in *C. gynandra* compared to the other two leafy vegetables. On the other hand, *B. oleracea* and *B. vulgaris* had the highest sinapic acid and ferulic acid content, respectively. In fact, the sinapic acid concentration in *B. oleracea* was 27 times more than that of *C. gynandra*.

### Antioxidant activity

A dose-dependent increase in radical scavenging activity was demonstrated by the three vegetable extracts (Table 1). Across the three tested concentrations (3.125, 50, and 200 μg/mL), *C. gynandra* exhibited a significantly high DPPH radical scavenging activity compared to both *B. oleracea* and *B. vulgaris*. The radical scavenging activity of *B. oleracea* was consistently the lowest at all tested concentrations.

**Figure 5:**
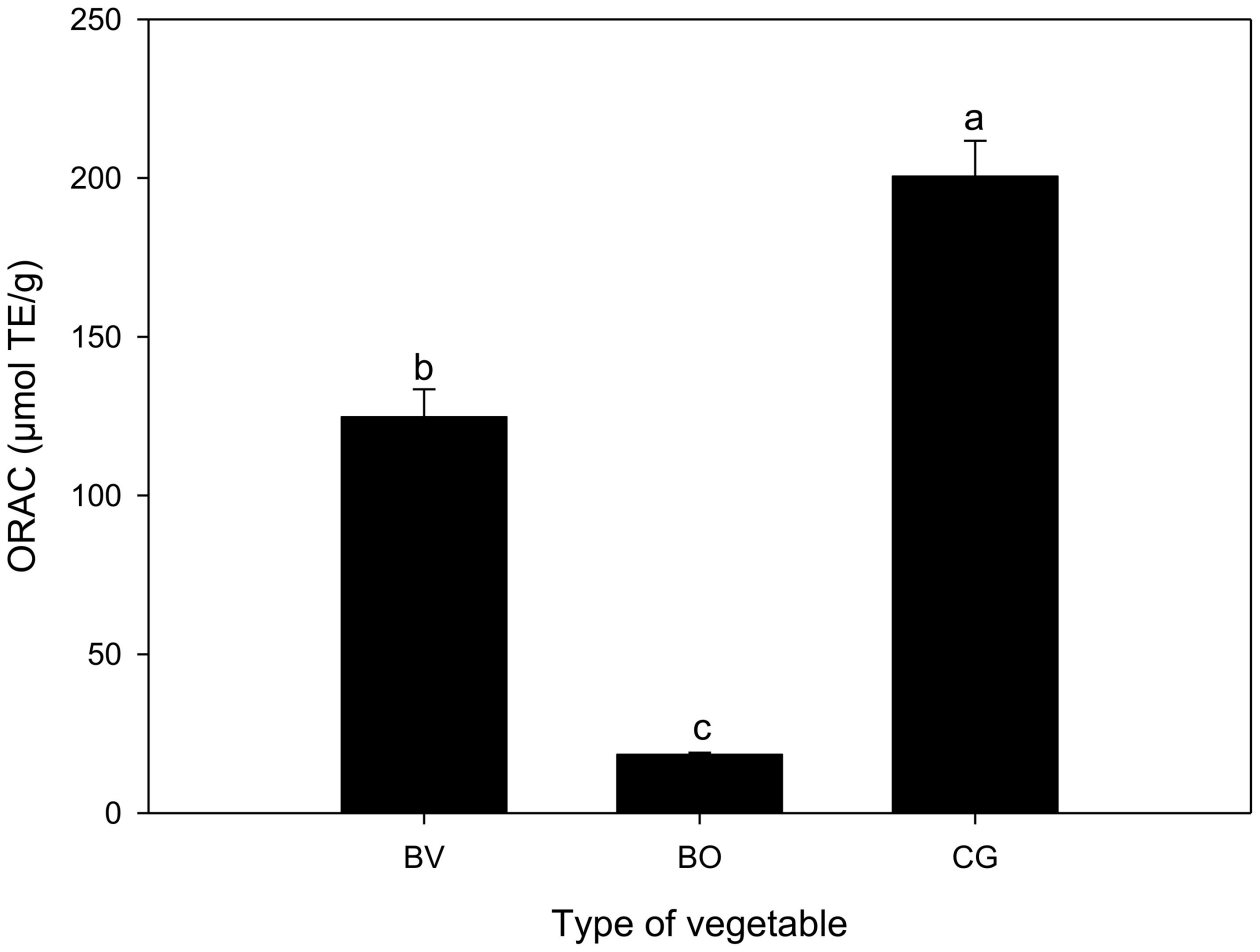
Oxygen radical absorbance capacity (ORAC, μmol TE/g DW) of extracts of *Beta vulgaris* (BV), *Brassica oleracea* (BO) and *Cleome gynandra* (CG). Data are mean ± standard deviation (*n* = 3). In each graph, bars with different letter are significantly different based on Duncan's multiple range test (*P* ≤ 0.05). Data analysis was performed using SPSS (Version 16).

Based on the oxygen radical absorbance capacity model (Figure 5), *C. gynandra* extract had the highest ORAC (200.57 μmol TE/g). This activity was 11- and 1.6-fold greater than *B. oleracea* and *B. vulgaris*, respectively. Overall, *C. gynandra* exhibited higher antioxidant activity in both the DPPH and ORAC model systems used in this study.

## Discussion

The search for alternative nutritious foods may hold the key to nourishing many people on the African continent and beyond. Wild green vegetables have been found to be rich sources of vitamins, namely β-carotene, ascorbic acid, riboflavin, folate as well as minerals such as iron, calcium and phosphorous (Gupta et al., 2005). The incorporation of wild food plants into the diet can introduce variety and diversity in terms of nutrition and taste (Cernansky, 2015). It is common knowledge that a diverse range of foods including vegetables is required to provide all the necessary micronutrients and phytochemical constituents for a healthy diet. Hence, dietary diversification is critical in order to mitigate the widespread nutrient deficiencies of vitamin A, iron, and zinc (FAO/WHO, 2004). The dietary requirement for a micronutrient refers to the intake level, which meets specified criteria for adequacy; however, threshold levels remain undefined for most dietary nutrients (FAO/WHO, 2004). The nutrient content of plant foods such as green leafy vegetables may vary widely due to several factors, which include stage of maturity and post-harvest handling (van Jaarsveld et al., 2014). In the present study, the vegetables were obtained in a fresh state at the point of sale in order to determine the nutritional and phytochemical properties of the quality available to consumers. Both *C. gynandra* and *B. vulgaris* exhibited higher concentrations of all the quantified nutrient elements compared to *B. oleracea*. *B. vulgaris* had higher levels of sodium, magnesium, manganese and copper, whereas phosphorus, potassium, calcium, iron and zinc content was higher in *C. gynandra*. This implies that a diverse diet incorporating both vegetables may be needed to provide adequate levels of the desired nutrients. Considerably high iron content in some wild, traditional leafy green vegetables was also recently reported (Schönfeldt and Pretorius, 2011; van Jaarsveld et al., 2014). Iron plays a critical role as an oxygen carrier from lungs to body tissues, a transport medium for electrons within cells and as an integral part of important enzyme systems such as cytochromes (Wessling-Resnick, 2000). Worldwide, but particularly in developing countries, iron deficiency is probably the most widespread nutritional deficiency disorder (Hallberg, 2001). Similar to our findings, some recent studies have reported underutilized wild leafy vegetables to be good sources of calcium (Gupta et al., 2005; van Jaarsveld et al., 2014). Calcium, an essential mineral comprising 1.9% of the body weight provides rigidity to the skeleton, and is vital in neuromuscular function, enzyme-mediated processes and blood clotting (WHO, 2009). Globally, calcium intake levels per capita vary widely and increase from developing countries, particularly Asia to developed countries such as North America (FAO/WHO, 2004). In the present study, zinc content was 1.2 and 2.1 times greater in *C. gynandra* compared to *B. vulgaris* and *B. oleracea*, respectively. Similarly, high zinc content in *C. gynandra* was reported in a study comparing five wild green vegetables (Schönfeldt and Pretorius, 2011). Zinc plays an important role in more than 300 enzymes involved in synthesis and degradation of biomolecules, metabolism of other micronutrients as well as the immune system (MacDonald, 2000). Similar to iron, cooking has no adverse effect on zinc levels in green leafy vegetables (Uusiku et al., 2010). However, bioavailability of zinc may be adversely affected by anti-nutritional factors such as phytic acid (WHO, 2009). Oxalic acid is another anti-nutritional factor, which interferes with calcium absorption by forming insoluble complexes (Gupta et al., 2005). Future studies on nutritional composition of wild green leafy vegetables should evaluate the content of these anti-nutritional compounds.

Vitamin A, an essential nutrient in humans, provides a vital role in the functioning of the visual system, and maintenance of cell function for growth and epithelial cellular integrity as well as production of red blood cells (WHO, 2009). Preformed retinol (mainly as retinyl ester) and provitamin A carotenoids such as β-carotene, lutein, violaxanthin, and neoxanthin are the main sources of dietary vitamin A (Blomhoff et al., 1991). In the present study, β-carotene content in *B. vulgaris* and *C. gynandra* was 28- and 22-times greater when compared to *B. oleracea*, respectively. With regards to plant sources, levels of vitamin A are dependent on β-carotene content, its bioavailability and bio-efficacy (West et al., 2002). Based on WHO (2009) recommendation, dietary diversification through the consumption of provitamin A-rich foods, namely dark green leafy vegetables is one of the three strategies to curb prevalence of vitamin A deficiency. The vitamin C content of *C. gynandra* was 4.5- and 3-times higher in comparison to *B. oleracea* and *B. vulgaris*, respectively. In a study by Pennington and Fisher (2010), dark green leafy vegetable subgroup (which included *B. vulgaris* and eight other commercial leafy vegetables) provided at least 50% of dietary reference intakes (DRIs) for vitamin C. A substitution of *B. vulgaris* with *C. gynandra* or an inclusion of *C. gynandra* in this subgroup will certainly amplify the percentage value for vitamin C DRIs. Besides its role as an electron donor for enzymes involved in collagen hydroxylation, carnitine biosynthesis and tyrosine metabolism, vitamin C is a potent antioxidant (Prockop and Kivirikko, 1995). Thus, the significantly high antioxidant activity observed in *C. gynandra* may correspond to its significant high level of vitamin C in comparison to other vegetables in the present study.

Apart from vitamins, non-nutritional components of wild green vegetables, notably phenolic compounds are also known to possess powerful radical scavenging properties against reactive oxygen species (ROS) (Stangeland et al., 2009). Total phenolic and flavonoid contents as well as specific phenolic compounds (hydroxybenzoic and hydroxycinnamic acid derivatives) were quantified in the three leafy vegetables. Notably, the concentration of protocatechuic acid, *p*-hydroxybenzoic acid, salicylic acid, caffeic acid, and *p*-coumaric acid were significantly higher in *C. gynandra* compared to the two widely consumed commercial vegetables. Oxidative stress is generally associated with the occurrence of numerous health conditions including neurodegenerative disorders, cardiovascular diseases and, most recently cancers (FAO/WHO, 2004). In the present study, *C. gynandra* exhibited the best antioxidant activity using both the DPPH and ORAC model systems. In particular, the ORAC affords a robust antioxidant test system, which measures both hydrophilic and lipophilic chain-breaking antioxidant capacity (Prior et al., 2003). Compared to synthetic antioxidant supplements, natural antioxidants derived from plant products such as green leafy vegetables may be more effective in reducing ROS levels due to the synergistic actions of a wide range of constituent biomolecules such as vitamins C and E, phenolic compounds, carotenoids, terpenoids, and phytonutrients (Podsedek, 2007). Regular intake of dietary antioxidants from phenolic compound-rich vegetables can reduce the adverse risk of these lifestyle-related diseases. Perhaps the reported biological activities of *C. gynandra* (Hamill et al., 2003; Muchuweti et al., 2007; Bala et al., 2010) are an emergent property of its high total phenolic and flavonoid content. However, there is a lack of documentation regarding the antioxidant status of the sub-Saharan Africa leafy vegetable population (Uusiku et al., 2010). Therefore, in most countries this area requires further scientific studies to establish baseline health data that can be used for better-targeted interventions. It is a paradox that such a plant diversity-rich continent is still characterized as food insecure and undernourished (FAO, 2014) in the twenty-first century.

## Conclusions

For a generation, Africa has been depicted as a hunger-stricken continent, stereotyped in the international media by imagery of malnourished children. The consumption of wild leafy greens or vegetables holds immense potential in nourishing many on the continent and beyond. Results of the current study on the chemical, nutritional and antioxidant properties of *C. gynandra*, a popular wild leafy vegetable in many parts of sub-Saharan Africa, provides compelling scientific evidence of its potential in adding diversity to the diet and contributing toward the daily nutritional requirements of millions of people. However, the realization of this “dream” depends on a number of factors, which include significant investments in the research on under-utilized foods, in particular wild leafy vegetables. Chemical constituents, nutritional and antioxidant qualities of *C. gynandra* provide critical insights about its potential in adding diversity to diets in sub-Saharan Africa and beyond. Thus, *C. gynandra* and other indigenous leafy vegetables may provide the ultimate weapon against dietary deficiencies.

## Author contributions

MM conceived research idea and prepared draft manuscript. MM, SOA, AOA, JG, MS, MJ, PT, and KD performed analysis of bioactive compounds and edited the manuscript.

### Conflict of interest statement

The authors declare that the research was conducted in the absence of any commercial or financial relationships that could be construed as a potential conflict of interest.

## Abbreviations

AAPH: 2,2-Azobis(2-methylpropionamidine) dihydrochloride
CE: Catechin equivalents
DPPH: 2,2–Diphenyl−1–picryl hydrazyl
DW: Dry weight
FAO: Food and Agriculture Organization of the United Nations
GAE: Gallic acid equivalents
HPLC-PDA: High-performance liquid chromatography- photodiode array
ICP-MS: Inductively coupled plasma mass spectrometry
IFAD: International Fund for Agricultural development
ORAC: Oxygen radical absorbance capacity
ROS: Reactive oxygen species
RSA: Radical scavenging activity
UHPLC: Ultra-high performance liquid chromatography
WHO: World Health Organization.
